# P-180. Tick-borne Encephalitis (TBE) in International Travelers and TBE Vaccine Recommendations for Travelers to Europe

**DOI:** 10.1093/ofid/ofaf695.404

**Published:** 2026-01-11

**Authors:** Rishi Srinivasan, Frederick Angulo, Stephanie A Duench, Alexander Davidson, Patrick Kelly, Mark Riddle, Kate Halsby, Andreas Pilz, Robert Steffen, James H Stark

**Affiliations:** Pfizer Vaccines, Boston, Massachusetts; Pfizer Vaccines, Boston, Massachusetts; Pfizer, Collegeville, Pennsylvania; Pfizer Vaccines, Boston, Massachusetts; Pfizer Vaccines, Boston, Massachusetts; Pfizer, Inc., New York, New York; Pfizer Inc, London, England, United Kingdom; Pfizer Corporation Austria, Vienna, Wien, Austria; University of Zurich, Epidemiology, Biostatistics and Prevention Institute, WHO Collaborating Centre for Travellers’ Health, Zurich, Switzerland; Department of Epidemiology, Human Genetics and Environmental Sciences, University of Texas, School of Public Health, Houston, Texas, USA, Zurich, Zurich, Switzerland; Pfizer Biopharma Group, Collegeville, Pennsylvania

## Abstract

**Background:**

Tick-borne encephalitis (TBE) is a potentially life-threatening infectious disease caused by the tick-borne encephalitis virus (TBEV). There are 25 European countries with TBE endemic areas (Figure 1). In recent years, the incidence of surveillance-reported TBE cases has increased in Europe and TBE endemic areas have expanded in Europe. TBE is preventable through vaccination. We summarized the published literature on travel-associated TBE cases and country-specific TBE vaccine recommendations for travelers.

**Figure:**
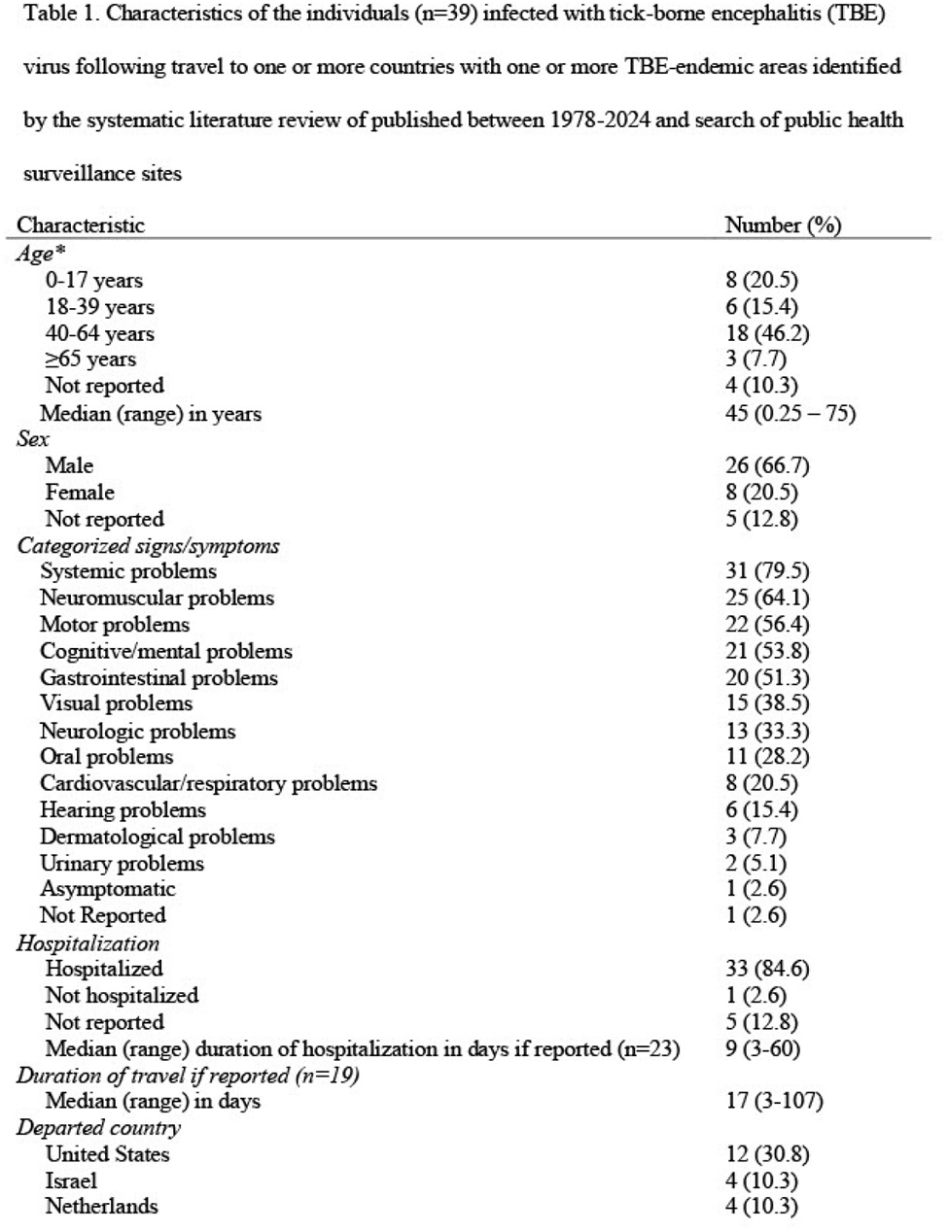

**Figure:**
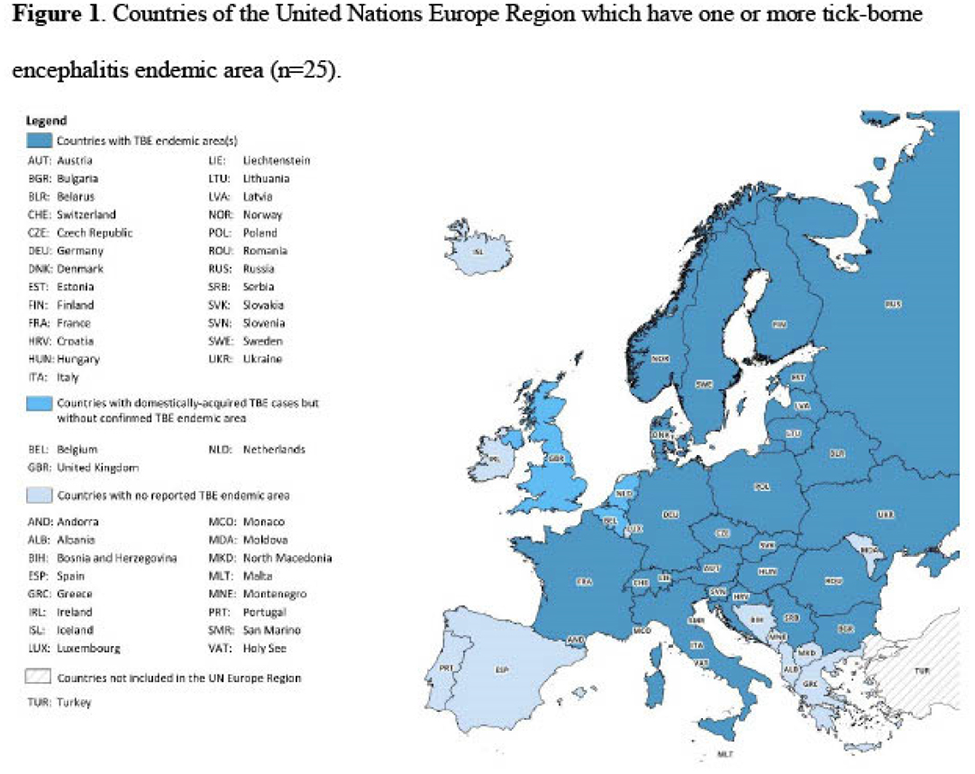

**Methods:**

We conducted a systematic literature review using PubMed and Web of Science and reviewed public health surveillance online sources to identify reports of travel-associated TBE cases published from 1978-2024. We also analyzed the European Centre for Disease Prevention and Control TBE Annual Epidemiological Reports from 2015-2022 for travel-associated cases and collected information on TBE vaccination recommendations by national public health authorities for travelers in Europe.

**Figure:**
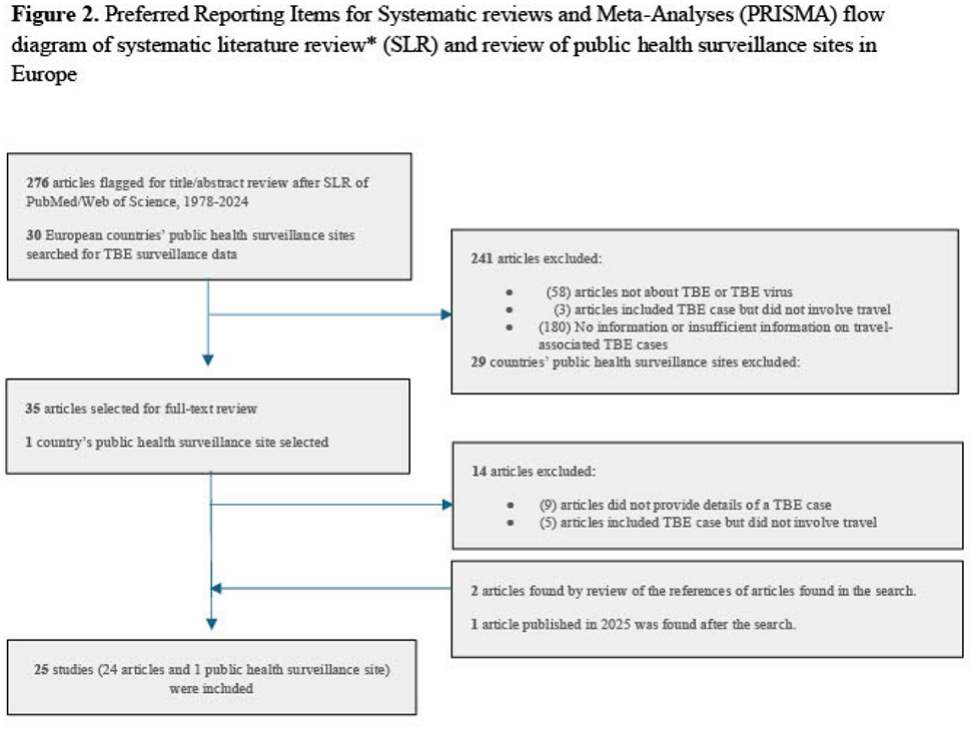

**Figure:**
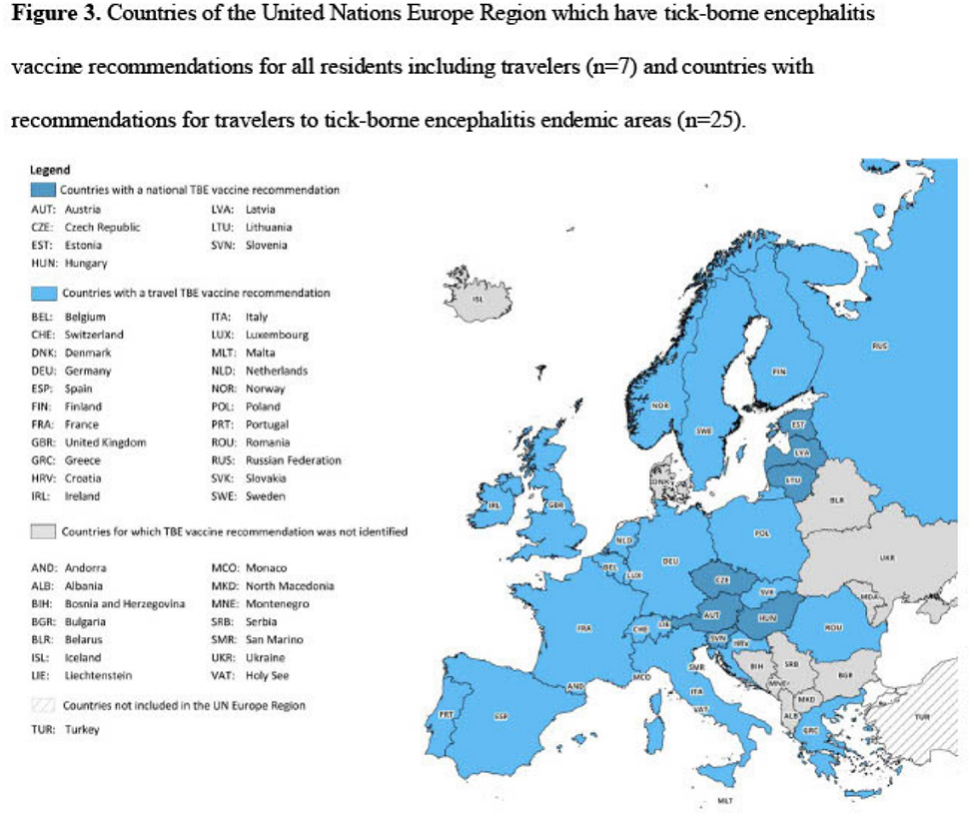

**Results:**

We identified 39 travel-associated TBE cases from 24 published articles and one public health report (Figure 2). The median age of the cases was 45 years; 26 (66.7%) were male (Table 1). Of the 33 travel-associated TBE cases with clinical information, 32 (97.0%) were hospitalized (median 8.5 days); one died. Travel-associated cases departed from the United States, Israel, and several countries in Europe; most frequently visited countries were Austria, Russia, Sweden, and Switzerland. Of the 22,191 surveillance-reported TBE cases reported to ECDC from 2015 to 2022, 376 (1.7%) were travel-associated cases. TBE vaccination recommendations for travelers were identified in 32 countries; seven countries recommend vaccination for all residents (including travelers) and 25 recommend vaccination for travelers to TBE endemic areas (Figure 3).

**Conclusion:**

Despite recommendations for TBE vaccination for travelers, travel-associated TBE cases among travelers to Europe continue to occur. Most of the published travel-associated TBE cases are associated with severe clinical illness. When considering the increasing geographic spread of the TBE endemic areas and increasing TBE incidence in Europe, enhanced efforts are needed to vaccinate travelers to TBE endemic areas in Europe.

**Disclosures:**

Rishi Srinivasan, BS, Pfizer Vaccines: Employer Frederick Angulo, DVM PhD, Pfizer Vaccines: Stocks/Bonds (Public Company) Stephanie A. Duench, PhD, Pfizer Vaccines: Stocks/Bonds (Public Company) Alexander Davidson, MPH, Pfizer Vaccines: Stocks/Bonds (Public Company) Patrick Kelly, PhD, Pfizer Vaccines: Stocks/Bonds (Public Company) Mark Riddle, MD, DrPH, Pfizer Vaccines: Employer|Pfizer Vaccines: Stocks/Bonds (Public Company) Kate Halsby, PhD, Pfizer Vaccines: Employer|Pfizer Vaccines: Stocks/Bonds (Public Company) Andreas Pilz, PhD, Pfizer Vaccines: Employer|Pfizer Vaccines: Stocks/Bonds (Public Company) Robert Steffen, MD, Pfizer Vaccines: Advisor/Consultant James H. Stark, PhD, Pfizer Vaccines: Employer|Pfizer Vaccines: Stocks/Bonds (Public Company)

